# Current landscape and tailored management of immune-related adverse events

**DOI:** 10.3389/fphar.2023.1078338

**Published:** 2023-03-06

**Authors:** Wenhui Liu, Zhiying Luo, Yiping Liu, Bao Sun

**Affiliations:** ^1^ Department of Pharmacy, The Second Xiangya Hospital, Central South University, Changsha, China; ^2^ Institute of Clinical Pharmacy, Central South University, Changsha, China; ^3^ National Clinical Research Center for Metabolic Diseases, The Second Xiangya Hospital, Central South University, Changsha, China

**Keywords:** immune checkpoint inhibitors, immune-related adverse events, clinical features, risk factors, mechanism-based strategies

## Abstract

Unprecedented advances have been made in immune checkpoint inhibitors (ICIs) in the treatment of cancer. However, the overall benefits from ICIs are impaired by the increasing incidence of immune-related adverse events (irAEs). Although several factors and mechanisms have been proposed in the development of irAEs, there is still incomprehensive understanding of irAEs. Therefore, it is urgent to identify certain risk factors and biomarkers that predict the development of irAEs, as well as to understand the underlying mechanisms of these adverse events. Herein, we comprehensively summarize the state-of-the-art knowledge about clinical features and the related risk factors of irAEs. Particularly, we also discuss relevant mechanisms of irAEs and address the mechanism-based strategies, aiming to develop a tailored management approach for irAEs.

## 1 Introduction

Immune checkpoint inhibitors (ICIs) therapy has revolutionized the management of multiple cancers and is now a mainstay approach for treating them. ICIs, including cytotoxic T lymphocyte protein 4 (CTLA-4), programmed cell death 1 (PD-1), and programmed cell death ligand 1 (PD-L1), mainly target proteins that negatively regulate T cell-meditated host immune response to cancer, thus enabling immune activation and antitumor response (Haslam and Prasad, 2019). Although patients in increasing number with cancer are eligible for ICIs therapy (Haslam et al., 2020), ICIs are also associated with a broad spectrum of side effects, termed as immune-related adverse events (irAEs) (Robert, 2020). These irAEs mirror aspects of primary autoimmune diseases and affect almost all organ systems (e.g., gastrointestinal tract, endocrine glands, skin and liver) with a range of severity (Dougan, 2017; Wang D. Y. et al., 2018; Postow et al., 2018; Liu et al., 2021a). Notably, irAEs are often accompanied with inflammatory side effects (Dougan, 2017), which are the important limitations to developing novel treatments.

It is worth noting that there are several aspects of irAEs that we have not yet understand, which has aroused several controversies in the field. For instance, several studies have indicated that there is a positive association of irAEs development with anti-tumor responses to ICIs or survival outcomes (Maher et al., 2019; Eggermont et al., 2020; Shankar et al., 2020). While other studies have supported poorer outcomes among patients with early or specific irAEs (Suresh and Naidoo, 2020). The possible reason is the difference of adjudication and attribution of irAEs, as well as immortal-time bias (Dall'Olio et al., 2020). Perhaps the management of irAEs is the realm that generates the greatest difficulties and controversies. Given that the anti-tumor responses and irAEs are representative of a robust immune reaction, corticosteroids or other immunosuppressive agents to control irAEs may lead to worse outcomes (Arbour et al., 2018).

As the administration of ICIs continues to increase in routine clinical practice, tailored management of irAEs has gained extensive attention. Current therapeutic recommendations against irAEs related to ICIs include discontinuation of immunotherapy and use of high-dose steroids. Meanwhile, American Society of Clinical Oncology (ASCO) (Brahmer et al., 2018), National Comprehensive Cancer Network (NCCN) (NCCN, 2021), Society for Immunotherapy of Cancer (SITC) (Brahmer et al., 2021) and European Society for Medical Oncology (ESMO) (Haanen et al., 2017) have also discussed the recommendations for specific irAE management. However, there is a need of further structured research to better identify the increased risk for irAEs and develop more individualized therapeutic strategies. In the present review, we highlight the current knowledge about clinical features and risk factors of irAEs, as well as address the mechanism-based strategies to develop a tailored management approach for these adverse events.

## 2 Clinical features, risk factors and potential biomarkers of irAEs

### 2.1 Clinical features of irAEs

Due to the emerging use of ICIs for the treatment of cancer, the cumulated number of irAEs is increasing exponentially. irAEs have been observed in almost all organ system, including the gastrointestinal, cutaneous, pulmonary, neurologic, genitourinary, cardiovascular, and integumentary systems, with the median onset of 2–16 weeks from the commencement of therapy (Weber et al., 2015). Moreover, irAEs are graded using the Common Terminology Criteria for Adverse Events (CTCAE) and range from grade 1 to grade 5, which refers to mild, moderate, severe, life-threatening or fatal events (National Cancer Institute, 2019) (Table 1). Onset of irAEs ranged from a few days of ICI initiation to over 1 year after completion of therapy (Yoest, 2017; Parakh et al., 2018), and the risk of first-onset irAEs was threefold lower in the time period between 4 weeks and the end of treatment than during the first 4 weeks of treatment (Kanjanapan et al., 2019). For instance, the dermatological toxicities were observed at approximately 2–3 weeks, gastrointestinal and hepatic toxicities at 6–7 weeks, and endocrinologic events at 9 weeks after treatment initiation of ipilimumab (Weber et al., 2012). Noteworthily, irAEs presumably persisted long after the cessation of treatment, and it was possible that irAEs might last for many years after treatment. A recent case indicated that autoimmune hepatitis occurred 8 months after discontinuation of nivolumab in a patient with metastatic melanoma (Parakh et al., 2018). More importantly, the development of characteristic irAEs was positively associated with improved patient outcome (Maher et al., 2019; Eggermont et al., 2020; Shankar et al., 2020). A multicenter study reported an increase in overall survival in patients with related irAEs compared with those with no related irAEs (Maher et al., 2019). Likewise, another multicenter study demonstrated that there was a positive association between multisystem irAEs and improved survival in patients with advanced non-small-cell lung cancer (NSCLC) treated with ICIs (Shankar et al., 2020). Eggermont et al. revealed that patients with stage III melanoma who developed irAEs had a longer recurrence-free survival (RFS) (Eggermont et al., 2020). Given the immune mechanisms of anti-PD-1/PD-L1 antibodies, it was reasonable to associate the occurrence of autoimmune events with improved prognosis, as activation of the immune system can lead to tumor responses and autoimmunity. However, current evidence suggested that the early or specific irAEs were correlated with worse survival in ICI-treated patients with NSCLC. The reasonable explanation might be the difference of adjudication and attribution of irAEs, as well as immortal-time bias (Dall'Olio et al., 2020).

**TABLE 1:** Clinical features of ICIs-related irAEs.

Cancer type	ICIs	Target	ICIs-related irAEs		References
			Any^a^	Grade 3–5^a^	
Genitourinary malignancies	Nivolumab (0.3, 2 or 10 mg/kg), atezolizumab (1200 mg), durvalumab (10 mg/kg) and pembrolizumab (200 mg)	Anti-PD1 and anti-PDL1	NA	Approximately 3%–15%	Maughan et al. (2017)
Advanced solid tumors	BMS-936558 (0.1, 0.3, 1.0, 3.0, or 10.0 mg/kg)	Anti-PD1	41%	14%	Topalian et al. (2012)
Advanced melanoma	Pembrolizumab, nivolumab and ipilimumab	Anti-PD1 and anti-CTLA-4	43.2%	5.4%	Elkrief et al. (2019)
Metastatic renal cell carcinoma	Nivolumab (0.3 mg/kg)	Anti-PD1	75%	5%	Motzer et al. (2015)
Metastatic renal cell carcinoma	Nivolumab (2 mg/kg)	Anti-PD1	67%	17%	Motzer et al. (2015)
Metastatic renal cell carcinoma	Nivolumab (10 mg/kg)	Anti-PD1	78%	13%	Motzer et al. (2015)
Advanced melanoma	Nivolumab (1 mg/kg) and ipilimumab (3 mg/kg)	Anti-PD1 and anti-CTLA-4	48.3%	93.8%	Lebbé et al. (2019)
Advanced melanoma	Nivolumab (3 mg/kg) and ipilimumab (1 mg/kg)	Anti-PD1 and anti-CTLA-4	33.9%	85.6%	Lebbé et al. (2019)
Metastatic melanoma	Pembrolizumab and nivolumab	Anti-PD1	NA	14%	Vesely and Chen (2020)

^a^ irAEs, are graded using the Common Terminology Criteria for Adverse Events (CTCAE) and mainly include rash, diarrhea, pneumonitis, colitis, nephritis, hypothyroidism, hepatitis, thyroiditis, *etc.* ICIs: Immune checkpoint inhibitors; irAEs: immune-related adverse events; PD-1: programmed cell death 1; CTLA-4: cytotoxic T lymphocyte protein 4; PD-L1: programmed cell death ligand 1; NA: not available.

A recent study reported that the incidence of all grade irAEs from ICI monotherapy was as high as 90% (Pauken et al., 2019). irAEs were first identified after the introduction of anti-CTLA therapy in clinical practice (Wolchok et al., 2010; June et al., 2017). Previous studies indicated an overall incidence over 70% with anti-CTLA-4 and 27%–78% with anti-PD-1/anti-PD-L1 agents (Topalian et al., 2012; Kumar et al., 2017; Maughan et al., 2017; Elkrief et al., 2019; Wang et al., 2019). Among the diverse irAEs, cutaneous toxicities including rash, pruritus, and vitiligo were the most frequent (Weber et al., 2012). The overall incidence of cutaneous toxicities stemmed from CTLA-4 blockade ranged from 37% to 70% for all-grade and 1%–3% for grade 3 or higher cutaneous toxicities (Hodi et al., 2010; Eggermont et al., 2015). Whereas, cutaneous toxicities were less frequently reported with anti–PD-1 agents (17%–37%). Of note, ICIs could result in dose-dependent toxicities. Motzer et al. found that the incidence was 75%, 67% and 78% for all grade irAEs in patients with metastatic renal cell carcinoma receiving nivolumab 0.3, 2, and 10 mg/kg, respectively, and 5%, 17% and 13% for grade 3 or 4 irAEs (Motzer et al., 2015). Furthermore, a phase IIIb/IV study involving 360 advanced melanoma patients determined that the incidence of grade 3 or 4 irAEs was 33.9% with nivolumab 3 mg/kg plus ipilimumab 1 mg/kg *versus* 48.3% with nivolumab 1 mg/kg plus ipilimumab 3 mg/kg, while the incidence of all grade irAEs was 85.6% *versus* 93.8% (Lebbé et al., 2019). Anti-PD-1 therapy was associated with a lower rate of irAEs compared with anti-CTLA-4 therapy; however, other studies discovered that there were several treated patients (ranging from 7% to 24%) with severe or even fatal (grade 2 or higher) irAEs (Baumeister et al., 2016; Marrone et al., 2016). Moreover, a recent clinical study with metastatic melanoma patients revealed that 14% of patients experienced severe (grade 3 or 4) toxicities with PD-1/PD-L1 inhibitors alone (Vesely and Chen, 2020). The major fatal irAEs included cardiotoxicity, neurotoxicity and interstitial pneumonia, with the incidence of as high as 45% (Wang D. Y. et al., 2018). A recent review demonstrated that myocarditis, a type of fatal irAEs, was reported in up to 3% of patients treated with anti-CTLA-4 but less than 1% of patients receiving PD-L1 inhibitors (Dougan and Pietropaolo, 2020). Another study found high neurotoxicity in advanced melanoma patients after nivolumab, ipilimumab, or combination treatment with an overall rate of 2.8% and in combination treatment with a rate of 14% (Spain et al., 2017). Furthermore, a recent study highlighted ICIs-related stevens-Johnson syndrome/toxic epidermal necrolysis (SJS/TEN) events as fatal irAEs (Ma et al., 2021). For these fatal irAEs, the mortality rate of ICI-related SJS was 19.9%, and the mortality rate of ICI-related TEN was 61.6% (Zhu et al., 2021). Besides, patients with advanced melanoma (stage III or IV) could experience long-term toxicities after termination of anti-PD-1, and up to 43.2% of patients developed chronic irAEs (defined as irAEs persisting for at least 12 weeks after anti–PD-1 therapy cessation) (Patrinely et al., 2021).

### 2.2 Risk factors or potential biomarkers of irAEs

The clinical features of irAEs are relatively obscure with subtle imaging changes and thus are difficult to determine, especially in the early stage. Therefore, it is imperative to understand and explore predictive risk factors or potential biomarkers for the occurrence of irAEs (Table 2).

**TABLE 2:** Risk factors or potential biomarkers of ICIs-related irAEs.

Risk factors or potential biomarkers	Cancer type	Treatment	Correlation with irAEs	References
*Risk factors*				
Pre-existing IBD	Solid tumors	Anti-PD1, anti-PDL1 or anti-CTLA-4	Increased the risk of gastrointestinal adverse events (diarrhea, colitis, nausea, and vomiting)	Abu-Sbeih et al. (2020)
Pre-existing autoimmune disease	Metastatic melanoma	Anti-PD1, anti-PDL1 or anti-CTLA-4	Increased the risk of *de novo* irAEs and deteriorated the existing autoimmune diseases	Abdel-Wahab et al. (2018), Michailidou et al. (2021)
*Potential biomarkers*				
*Immune cells*				
Increased WBC count and decreased RLC	Advanced melanoma	Nivolumab (2 mg/kg)	Increased the risk of severe irAEs and lung or gastrointestinal irAEs	Fujisawa et al. (2017)
Levels of NLR, PLR and neutrophil	Advanced NSCLC	Anti-PD1	Increased risk of severe irAEs	Liu et al. (2021a)
*Cytokines*				
Low baseline IL6 serum levels	Metastatic melanoma	Ipilimumab (3 mg/kg)	Increased the risk of irAEs	Valpione et al. (2018)
Cytokine levels	Solid tumors	Anti-PD1, anti-PDL1 and anti-CTLA-4	Associated with heightened risk of irAEs	Khan et al. (2019a)
Cytokine levels	Advanced melanoma	Pembrolizumab or nivolumab	Positively associated with severe irAEs	Lim et al. (2019)
Baseline IL-17 level	Advanced melanoma	Ipilimumab (10 mg/kg)	Positively correlated with severe diarrhea/colitis	Tarhini et al. (2015)
Elevated serum levels of IL-6	Malignant melanoma	Nivolumab	Increased the risk of psoriasiform dermatitis	Tanaka et al. (2017)
Increased eosinophils, IL-6, IL-10, and IgE	Solid tumors	Anti-PD1, anti-PDL1 or anti-CTLA-4	Increased risk of immune-related skin toxicities	Phillips et al. (2019)
Decreased levels of IL-10	Bladder cancer	Ipilimumab (3 mg/kg)	Positively associated with bilateral severe anterior uveitis	Sun et al. (2008)
*Genetic variability*				
HLA-DRB1	Physician-diagnosed rheumatoid arthritis	Anti–PD-1 or anti–PD-L1 therapy	Positively associated with ICI-induced inflammatory arthritis	Cappelli et al. (2019)
HLA-DR4	Solid tumors	Anti–PD-1 or anti–PD-L1 therapy	Developed autoimmune, insulin-dependent diabetes	Stamatouli et al. (2018)
rs4553808	Metastatic melanoma	Ipilimumab	Increased risk of endocrine irAEs	Queirolo et al. (2018)
rs2227981	NSCLC	Nivolumab (3 mg/kg)	Decreased incidence of any grade treatment-related toxicities	Bins et al. (2018)
*Gut microbiome*				
Increased *Bacteroidetes* phylum	Metastatic melanoma	Ipilimumab (3 mg/kg)	Positively associated with the resistance to immune-mediated colitis	Dubin et al. (2016)
Reduction of *Firmicutes*	Metastatic melanoma	Ipilimumab (3 or 10 mg/kg)	Positively associated with immune-mediated enterocolitis	Chaput et al. (2017)
Higher abundance of *Bacteroides*	Advanced melanoma	Ipilimumab in combination with nivolumab or pembrolizumab	Positively correlated with grade 3 irAEs	Andrews et al. (2021)

ICIs: Immune checkpoint inhibitors; irAEs: immune-related adverse events; IBD: inflammatory bowel disease; PD-1: programmed cell death 1; CTLA-4: cytotoxic T lymphocyte protein four; PD-L1: programmed cell death ligand 1; WBC: white blood cell; RLC: relative lymphocyte count; NSCLC: non-small-cell lung cancer; NLR: neutrophil-to-lymphocyte ratio; PLR: platelet-to-lymphocyte ratio, IgE, immunoglobulin E.

#### 2.2.1 Pre-existing autoimmune disease and physical parameters

Since irAEs are characterized by the abnormal activation of the immune system, patients with autoimmune disease may potentially have greater risks of developing irAEs. Retrospective studies indicated that patients with autoimmune diseases treated with ICIs had high rate (ranging from 28% to 60%) of having autoimmune disease flare (Danlos et al., 2018; Alexander et al., 2021; Fountzilas et al., 2022). A multicenter, retrospective study including 102 patients with underlying inflammatory bowel disease (IBD) treated with ICIs showed that gastrointestinal adverse events occurred in 41% of these patients compared with 11% of patients without histories of IBD (*p* < 0.001) (Abu-Sbeih et al., 2020). On the other hand, irAEs could also affect other new organ sites that were unaffected by autoimmunity prior to ICI therapy. A systemic review enrolling 123 patients with autoimmune diseases treated with ICIs indicated deterioration of existing autoimmune diseases in 50% of patients, *de novo* irAEs in 34% of patients, and both in 9% of patients (Abdel-Wahab et al., 2018). Another retrospective study analyzed 470 patients treated with ICIs and identified an association of the development of irAEs with both pre-existing history of autoimmune disease (adjusted OR = 2.57, 95% CI 1.46–4.52, *p* = 0.001) and family history of autoimmune disease (adjusted OR = 5.98, 95% CI 2.20–16.23, *p* < 0.001) (Michailidou et al., 2021). Inconsistently, a retrospective analysis of 417 patients treated with ICIs showed that there was no association between underlying autoimmune disease and irAE incidence or severity (Yeung et al., 2021). The possible explanation was the interindividual difference of patients’ status and risk for autoimmune disease at initiation of cancer immunotherapy.

In addition to the pre-existing autoimmune diseases, sex and body mass index was also identified as risk factors for the development of irAEs (Conforti et al., 2018; Valpione et al., 2018; Guzman-Prado et al., 2021). A recent meta-analysis revealed that higher BMI was associated with an increased risk of irAEs in patients on ICI therapies (OR = 2.62, 95% CI 1.70–4.03, *p* ≤ 0.00001) (Guzman-Prado et al., 2021). Moreover, a systematic review and meta-analysis reported that ICIs could improve overall survival for patients of both sexes, but males had a larger treatment effect from these drugs than females (Conforti et al., 2018). Another research supported that females were likely to be higher rates of irAEs (OR = 1.50, 95% CI 1.06–2.16 *p* = 0.022) (Valpione et al., 2018). On the contrary, ICIs-hypophysitis showed a more frequent occurrence in men than women, and the gender distribution of neurologic and vascular irAEs also appeared to be male-dominant (Triggianese et al., 2020). One of the possible explanations was that females were at higher risk of several autoimmune diseases. On the other hand, these differences might also be influenced by other variations such as genetic and ethnic factors. Further studies are needed to elucidate this disparity and the important roles of sex-specific factors in the development of irAEs.

#### 2.2.2 Specific immune cells and cytokines

Immune cells and cytokines play essential roles in tumor microenvironment and immune homeostasis (Harlin et al., 2009; Peng et al., 2012), which can be utilized by immunotherapy and contribute to the development of irAEs. In a multicenter study, Fujisawa et al. enrolled 101 patients with melanoma treated with nivolumab and revealed that increases in total white blood cell (WBC) count and decreases in relative lymphocyte count (RLC) were associated with severe irAEs (Fujisawa et al., 2017). Our previous study also concluded that levels of neutrophil-to-lymphocyte ratio (NLR), platelet-to-lymphocyte ratio (PLR) and neutrophil were associated with the increased risk of severe irAEs (Liu et al., 2021b). The possible explanation was that lymphopenia might suggest impaired cell-mediated immunity, whereas neutrophilia might reflect an intensive response to systemic inflammation (Ocana et al., 2017). In recent years, cytokine levels were also linked with the development of irAEs. Lower baseline levels and greater post-treatment increases in multiple chemokines were reportedly associated with irAEs (Khan S. et al., 2019). Emerging data showed that increased levels of proinflammatory cytokines, including IL1, IL2, and IFNα2, were strongly associated with severe irAEs, which could be integrated into the CYTOX score to predict severe irAE development (Lim et al., 2019). Tarhini et al. found that higher baseline IL17 levels were correlated with the development of grade 3 colitis in patients with melanoma treated with neoadjuvant ipilimumab (Tarhini et al., 2015). Intriguingly, Tanaka and others observed that increase in circulating IL-6 was significantly associated with irAEs in patients treated with nivolumab with psoriasiform dermatitis (Tanaka et al., 2017). Moreover, a retrospective analysis of 285 patients demonstrated that increased eosinophils, IL-6, IL-10, and immunoglobulin E were associated with immune-related cutaneous adverse events (Phillips et al., 2019). Mechanistically, elevated IL-6, as an autocrine regulator, promotes the development of Th17 cells and thus may enhance inflammation of the skin progressing to epidermal hyperplasia (Watanabe et al., 2009). Another prospective study observed that low baseline IL6 serum levels were associated with higher rates of irAEs in patients with metastatic melanoma treated with ipilimumab (Valpione et al., 2018). Similarly, a decrease in levels of IL-10 was reportedly associated with irAEs in patients treated with ipilimumab and anti-CTLA-4 ticilimumab (Reuben et al., 2006; Sun et al., 2008). These results are consistent with inference that IL6 increases tumor invasiveness and compromises the immune-inflammatory regulation (Patel and Gooderham, 2015; Roth et al., 2016), which may impair the immune response elicited by CTLA4 blockade and lower the risk of irAEs. Therefore, these findings collectively indicate the association of immune cells and cytokines with irAEs, which may be helpful in discovering potential biomarkers and practical therapeutic targets for irAEs.

#### 2.2.3 Genetic variability

Due to genetic factor as a contributing factor for autoimmunity, the role of genetic variability has also been implicated in the development of irAEs. A pilot study indicated that patients with ICI-induced inflammatory arthritis of European descent were more likely to be positive for HLA-DRB1 shared epitope alleles than healthy controls (Cappelli et al., 2019). Moreover, a striking predominance of HLA-DR4 occurred in patients treated with ICIs who developed autoimmune, insulin-dependent diabetes (Stamatouli et al., 2018). A cohort study including 173 patients with melanoma treated with ipilimumab identified that CTLA-4 SNP -1661A>G (rs4553808) was correlated with an increased risk of endocrine irAEs (Queirolo et al., 2018). Another analysis of 96 patients with NSCLC treated with nivolumab found an association of PDCD1 804 C>T (rs2227981) with decreased incidence of irAEs (Bins et al., 2018). These initial findings are required to be validated in the future studies with larger patient cohorts.

#### 2.2.4 Gut microbiome

Increasing evidence provided that gut microbiota, such as *Bacteroides*, *Clostridium* and *Faecalibacterium*, was implicated in the maintenance of immune homeostasis stimulating the production of anti-inflammatory cytokines or inducing expansion of T-regulatory cells, which might be also involved in the development of irAEs (Mazmanian et al., 2005; Sokol et al., 2008; Atarashi et al., 2011; Clavel et al., 2017; Gut Microbiota May Mediate AEs, 2021; Pham et al., 2021).

A prospective study claimed that increased abundance of bacteria belonging to the *Bacteroidetes* phylum was associated with a reduced incidence of colitis in metastatic melanoma patients treated with ipilimumab (Dubin et al., 2016). Notably, another clinical study showed that a significant reduction of *Firmicutes* (2 times lower than baseline) was correlated with ipilimumab-induced colitis in metastatic melanoma patients (Chaput et al., 2017). Recently, Andrews et al. profiled the gut microbiota signatures using 16s RNA sequencing in patients treated with PD-1 and CTLA-4 inhibitors, and demonstrated a significantly higher abundance of *Bacteroides* compared with those without irAEs (Andrews et al., 2021). Also, our real-word study provided that patients with severe irAEs showed a visibly higher abundance of *Streptococcus*, *Paecalibacterium*, and *Stenotrophomonas*, and patients with mild irAEs had a higher abundance of *Faecalibacterium* and unidentified_*Lachnospiraceae*, which jointly suggested that gut microbiota could serve as an informative source for developing predictive biomarkers and predicting the occurrence of irAEs (Liu et al., 2021c). A previous study highlighted that the microbial-derived products could trigger an innate immune response, eventually leading to the activation of self-reactive immune cells (Khan Z. et al., 2019), which might interpretate that patients with bacterial disorder were more likely to experience irAEs. Future prospective clinical trials should focus on the biomarkers based on pretreatment risk of irAEs for better prediction of ICI-related irAEs.

## 3 Relevant mechanisms of irAEs

**FIGURE 1:**
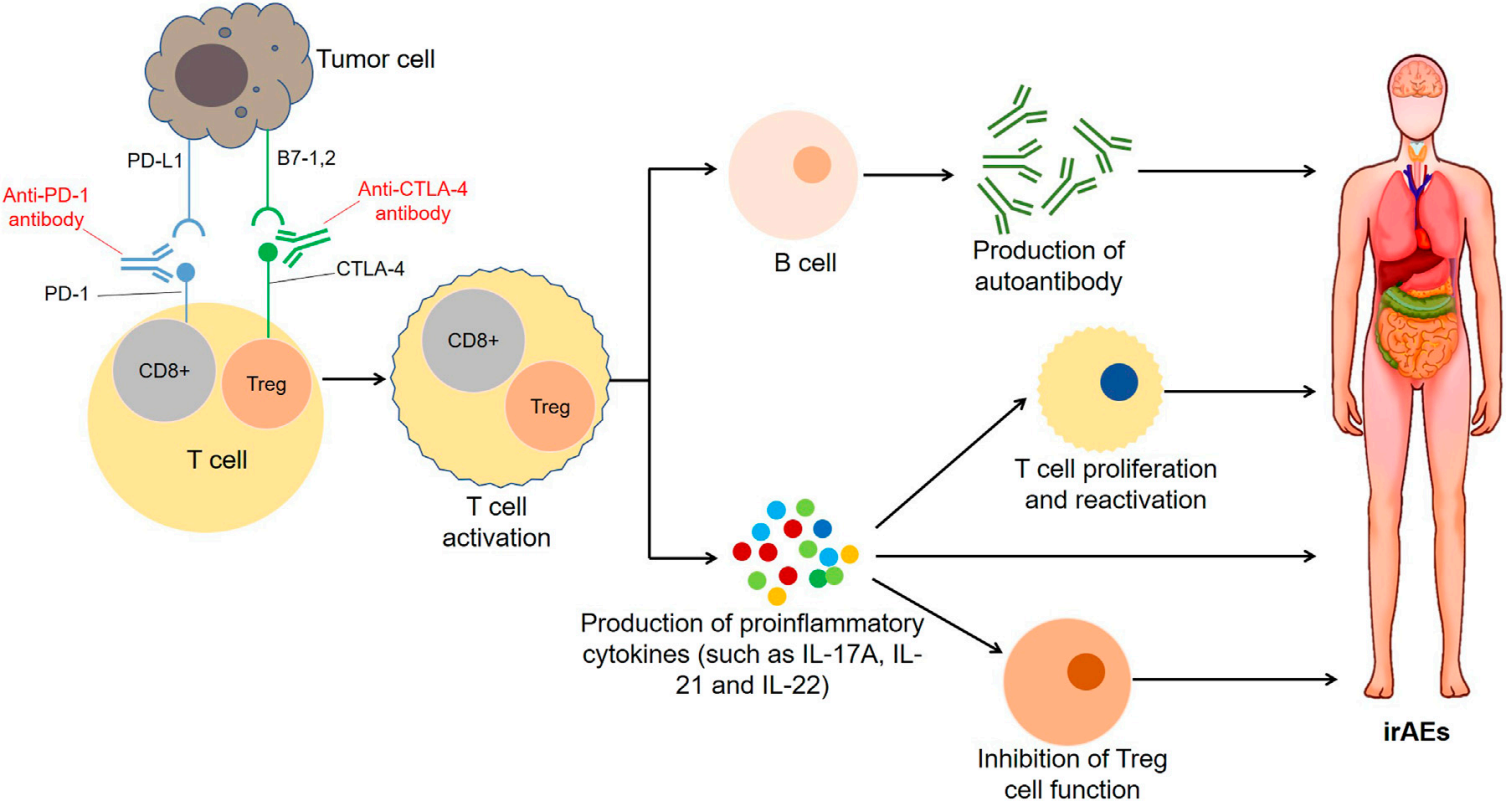
Relevant mechanisms of irAEs in response to ICIs. ICIs including PD-1, PD-L1 and CTLA-4 inhibitors, can inhibit immune checkpoints (PD-L1 and B7-1,2) and induce a series of cellular alterations, such as activated autoimmune CD8^+^ T cells and impaired Treg cell survival, and subsequently lead to T cell activation. On the one hand, activated T cell interacts with B cell, which can result in autoantibody production and ultimately lead to irAEs. On the other hand, T cell activation can induce the production of proinflammatory cytokines, such as IL-17A, IL-21 and IL-22, which consequently impaires the Treg cell function, induces T cell proliferation or reactivation and directly or indirectly contributes to irAEs.

Although there are certain commonalities between the irAEs and autoimmune diseases or autoinflammatory reactions, the mechanisms behind irAEs are not well understood. There are several proposed main mechanisms that contribute to irAEs in response to ICIs (Figure 1).

### 3.1 T cell activation or reactivation

Previous studies utilized a CTLA-4 knockout mice model and found that the mice developed T cell lymphoproliferation and T cell-mediated autoimmunity (Wing et al., 2008; Klocke et al., 2016). In accordance with these data in mice, individualsx with CTLA-4-related genetic disorders also had regulatory T (Treg) cell defects and autoimmune infiltration (Lo et al., 2016). Consistent with these results, several recent researches suggested that activation or reactivation of T cells was thought to be a dominant factor in the development of ICI-related irAEs (June et al., 2017; Dougan et al., 2021a). Recently, Takahashi et al. found that CD8^+^ lymphocyte infiltration was significantly greater in irAE colitis than that in ulcerative colitis (Takahashi et al., 2023). Further research confirmed that activated cytotoxic CD8^+^ T cells accelerated anti-PD-1 antibody-induced psoriasis-like dermatitis *via* IL-6 (Tanaka et al., 2020). Typically, Treg cells downregulate immune responses by inhibiting effector T cell proliferation and cytokine release to regulate self-tolerance. The functional homeostasis between Treg cells and type 17 T helper (Th17) cells served as a prominent factor in irAEs associated with ICIs (Knochelmann et al., 2018). Enhanced Th17 cell responses could contribute to the production of proinflammatory cytokines such as IL-17A, IL-21 and IL-22, consequently participating in the pathogenesis of autoimmune diseases and irAEs (Noack and Miossec, 2014; Tarhini et al., 2015).

Interestingly, a comprehensive review highlighted that cross-reactivity between anti-tumor T cells and similar antigens on healthy cells might also underlie the pathogenesis of some irAEs (Michot et al., 2016). For instance, vitiligo appeared to be more frequent in patients with melanoma, which suggested cross-reactivity between T cells and tumor antigens and melanocytes (Sandigursky and Mor, 2018). Due to the low selectivity among the tumor-reactive T cell subjects, cross-reactivity had also been suggested for ICI-related myocarditis \(Cheng and Loscalzo, 2017). Johnson et al. reported that two patients developed fatal myocarditis after treatment with ipilimumab and nivolumab (Johnson et al., 2016). Possible mechanism for this toxicity was that the tumor, skeletal muscle, and heart shared the common high-frequency T-cell receptors, which led to T-cell-driven drug reaction. In recent years, Berner et al. identified nine shared antigens between tumor tissue and skin, which was able to stimulate CD8^+^ and CD4^+^ T cells *in vitro*, highlighting a potential mechanism of ICI-mediated autoimmune toxic effects (Berner et al., 2019).

### 3.2 Cytokines production

In addition to the regulation of T cells, both anti-CTLA-4 and anti-PD-1/PD-L1 antibodies could increase cytokine production. Anti-CTLA-4 antibody could enhance CD4^+^ and CD8^+^ T cell activation, subsequently releasing cytokines including tumor necrosis factor (TNF), interferon-γ (IFNγ) and IL-2, which contributed to further T cell proliferation and reactivation (Laurent et al., 2013; Seidel et al., 2018). Besides, Luoma et al. observed the upregulated expression of cytokines and their receptors in both T cell and myeloid cell of ICI-related colitis patients, which suggested that other innate and T cell-derived cytokines were associated with ICI-related colitis and could serve as additional treatment targets (Luoma et al., 2020). In recent years, TNF inhibitors have been successfully adopted to treat different irAEs in patients receiving ICIs, further highlighting the importance of cytokines in the pathogenesis of irAEs (Kim et al., 2017). Fundamentally, further studies are required to address the precise roles of these cytokines in irAEs and the development of T cell activation or reactivation.

### 3.3 B cells and autoantibody production

Interactions between T cells and B cells can result in autoantibody production and play an important role in humoral immunity, while aberrant interactions are associated with autoimmunity (Petersone et al., 2018). Das et al. found B cell changes in patients with irAEs after the first cycle of therapy with either anti-CTLA4 or anti-PD1, or in combination, and they further identified B cell activation in cells with genomic profiles of CD21lo B cells by single-cell RNA sequencing, which implied that targeting B cells might reduce irAEs in these patients (Das et al., 2018). On the other hand, the development of autoantibodies was common with ipilimumab treatment, and autoantibody formation was associated with irAEs and antitumor efficacy (de Moel et al., 2019). Toi et al. indicated that the presence of the preexisting antibodies was independently associated with the development of irAEs in patients with NSCLC treated with anti-PD-1 (Toi et al., 2019). A recent study indicated that ICIs might exacerbate bullous pemphigoid (BP) as an irAE through the generation of anti-BP180-NC16A IgG autoantibodies (Sadik et al., 2019). Whether these autoantibodies reflect an underlying mechanism needs further elaborated experimental researches to validate.

## 4 Mechanism-based therapeutic strategies

Understanding the related mechanisms driving irAEs will contribute to targeted therapeutic strategies. Alongside the recommendations and therapeutic algorithms of ASCO [11], NCCN [12], SITC [13] and ESMO [14] for irAE management, several mechanism-based therapeutic strategies are also discussed (Table 3).

**TABLE 3:** Mechanism-based therapeutic strategies for irAEs.

Strategies	Subjects	Possible mechanisms	References
Glucocorticoids	Mice with irAEs	Inhibited antitumor T cell responses and induced apoptosis of activated T cell	Pauken et al. (2019)
Mycophenolate-containing immunosuppressants	Patients with steroid-refractory pneumonitis	Inhibited T cell reactivation	Thompson et al. (2019)
Fecal microbiota transplantation	Patients with refractory ICI-associated colitis	A substantial reduction in CD8^+^ T-cell density	Wang et al. (2018b)
Vedolizumab	Patients with ICI-induced enterocolitis or IMDC	Inhibited the migration of T cells	Bergqvist et al. (2017), Abu-Sbeih et al. (2018)
Infliximab	Patients with irEC	Inhibition of inflammatory cytokine	Johnson et al. (2018)
TNF inhibitors	Xenografted mice with human colon cancer cells	Inhibition of inflammatory cytokine	Perez-Ruiz et al. (2019)
Tocilizumab	Patients with irAEs	Decreased C-reactive protein	Stroud et al. (2019)
Infliximab	Patients with steroid-refractory irAEs	NA	Araujo et al. (2021)
Abatacept and alemtuzumab	Patients with immune-related myocarditis	Inactivation of the normal immune response	Salem et al. (2019), Esfahani et al. (2019b)
Secukinumab	Patients with ICI-induced inflammatory arthropathy	NA	Ma et al. (2022)
Immunoglobulin	Patients with neurological and hematological irAEs	Suppression of inflammation	Schwab and Nimmerjahn (2013)
Plasma exchange	Patients with myasthenia gravis or Guillain–Barré syndrome	Removed the pathogenetic autoantibody	Touat et al. (2017)
Rituximab	Patients with immunotherapy-induced myasthenia gravis	NA	Verma et al. (2021)

irAEs: immune-related adverse events; ICI: immune checkpoint inhibitor; IMDC: immune-mediated diarrhea and colitis; irEC: immune-related enterocolitis; NA: not available.

### 4.1 Inhibition of T cell response and migration

Given that activation or reactivation of T cells is considered to be the dominant factor in the development of ICI-related irAEs, inhibition of T cell response and migration may be a tailored strategy for irAEs. Glucocorticoids are the preferred treatment for most irAEs except for endocrine irAEs and effective in mitigating symptoms. Animal models demonstrated that glucocorticoids might inhibit antitumor T cell responses and were well known to induce apoptosis of activated T cell (Pauken et al., 2019). Ambiguously, there are some disparities in findings from retrospective studies of patients’ treatment with ICIs who received glucocorticoids, though it is suggested that they overall had deleterious effects on antitumor responses, particularly when glucocorticoids were used at initiation of immunotherapy (Arbour et al., 2018; Faje et al., 2018). Moreover, there is a need to introduce steroid-sparing strategies with the occurrence of steroid-refractory irAEs, mainly including immune-related hepatitis, nephritis and pancreatitis. For instance, patients with steroid-refractory pneumonitis can be treated with mycophenolate-containing immunosuppressants (Thompson et al., 2019). Due to the significant side effects in the treatment of ICI-associated colitis with corticosteroids or immunosuppressive agents, a recent study provided novel evidence that modulation of the gut microbiome *via* fecal microbiota transplantation could abrogate ICI-associated colitis with a substantial reduction in CD8^+^ T-cell density (Wang Y. et al., 2018). Recent studies reported that vedolizumab, an inhibitor of α4β7 integrin that inhibited the migration of T cells into gastrointestinal mucosa, could be used instead of infliximab against immune-related colitis (Bergqvist et al., 2017; Abu-Sbeih et al., 2018), proposing α4β7 integrin as an attractive target for immunotherapy toxicities.

### 4.2 Cytokine blockade

Owing to the important roles of acute inflammatory cytokines, such as TNFα, IL-6 and IL-1β in irAEs, inhibiting these cytokines may be a promising strategy for the treatment of irAEs. Of these, inhibition of TNFα appeared to be effective for severe, refractory, immune-related colitis and inflammatory arthritis (Baddley et al., 2018; Mooradian et al., 2020; Dougan et al., 2021b). Johnson et al. suggested that for grades 3 and 4 colitis patients, the addition of infliximab (targeted TNFα) to glucocorticoids was significantly associated with a shorter time to symptom resolution than utilization of glucocorticoids alone (Johnson et al., 2018). In a mice model, TNF inhibitors concomitantly with combined CTLA-4 and PD-1 immunotherapy ameliorated colitis and improved antitumor efficacy, which provided clinically feasible strategies to dissociate efficacy and toxicity for cancer immunotherapy (Perez-Ruiz et al., 2019).

### 4.3 Monoclonal antibodies

Monoclonal antibodies have also been recommended for the management of some steroid-refractory irAEs (Kim et al., 2017; Siakavellas and Bamias, 2018). Clinical improvement was noted in 80% of patients with nivolumab-associated grades 3 and 4 irAEs (predominantly pneumonitis) who received tocilizumab (targeted IL-6) (Stroud et al., 2019). Infliximab, a chimeric human-murine monoclonal antibody, was used to the treatment of steroid-refractory irAEs (Araujo et al., 2021). In recent years, a series of cases of successful response to monoclonal antibodies, such as abatacept (targeted CTLA-4) and alemtuzumab (targeted CD52), were reported in patients with immune-related myocarditis (Esfahani et al., 2019b; Salem et al., 2019). A recent case also demonstrated that secukinumab (targeted IL-17A) could effectively manage ICI-induced inflammatory arthropathy (Ma et al., 2022). Additionally, intravenous immunoglobulin comprised of pooled IgG antibodies from the serum of thousands of donors was considered as a second-line therapy for neurological and hematological irAEs (Schwab and Nimmerjahn, 2013). Since several irAEs were caused directly by autoantibodies, such as some hematological or neuromuscular irAEs, plasma exchange could be a feasible strategy against irAEs by removing the pathogenetic autoantibody from the circulation, especially in severe cases of myasthenia gravis or Guillain–Barré syndrome (Touat et al., 2017). For instance, Verma et al. showed that rituximab (targeted CD20) could be used to treat patients with immunotherapy-induced myasthenia gravis (Verma et al., 2021). Clinical trials evaluating strategies to prevent irAEs are needed to institute in the future.

Despite the benefits of tailored management for irAEs, early intervention and prophylaxis has grown in importance. A retrospective analysis of 179 patients with immune-related colitis of all grades found that compared with patients who received immunosuppressive therapy (infliximab or vedolizumab) > 10 days after onset of colitis, patients who received immunosuppression therapy early (≤10 days) were less likely to be hospitalized, experienced steroid taper failure less frequently, had a shorter course of steroid treatment and had a shorter duration of symptoms (Abu-Sbeih et al., 2019). The JAK-STAT and mTOR pathway played important roles in cellular processes and mediated downstream signaling of numerous cytokines. A previous studies indicated that excessive JAK1 signaling contributed to cancer evasion and development of autoimmunity (Chan et al., 2019), and inhibition of JAK-STAT displayed synergistic effects with ICIs to overcome treatment resistance (Lu et al., 2017). Furthermore, the addition of the mTOR inhibitor in a melanoma patient treated with PD-1 blockade could promote ongoing anti-PD-1 efficacy and reduce levels of irAE-related cytokines/chemokines (e.g., IL-5, IL-17, and CXCL10) (Esfahani et al., 2019a). There are also several prophylactic strategies through disease-specific therapy (such as vedolizumab for IBD) before commencing ICI to prevent toxicity in patients with underlying active autoimmune disease (Haanen et al., 2020b). Due to the fact that not all patients with autoimmune diseases experienced irAEs, prophylactic strategies might be considered and studied in prospective trials. Nevertheless, proactive commencement of disease-specific therapy was a reasonable strategy for patients with a high risk of new toxicity and few alternative treatment options (Haanen et al., 2020a).

## 5 Conclusion and future perspectives

**FIGURE 2:**
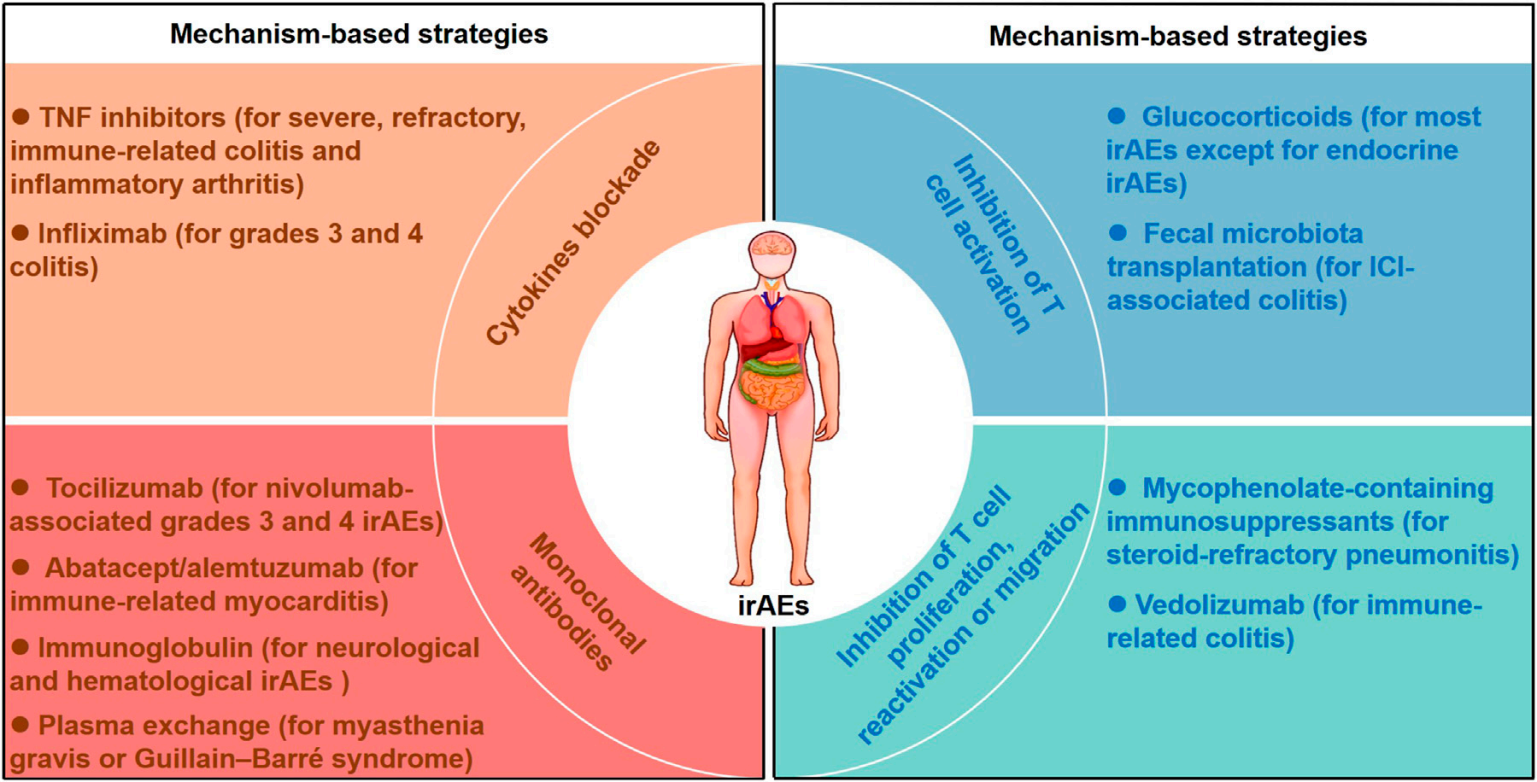
Mechanism-based therapeutic strategies for managing irAEs. Mechanism-based therapeutic strategies including inhibition of T cell activation, inhibition of T cell proliferation, reactivation or migration, cytokines blockade and monoclonal antibodies are listed. Glucocorticoids inhibit T cell activation for most irAEs except for endocrine irAEs, and fecal microbiota transplantation abrogates ICI-associated colitis with a reduction in CD8^+^ T-cell density. Vedolizumab and mycophenolate-containing immunosuppressants were used for steroid-refractory irAEs by inhibting T cell migration, proliferation or reactivation. Cytokines blockade including infliximab and TNF inhibtors were considered in severe, refractory and immune-related colitis. Monoclonal antibodies including tocilizumab, abatacept, alemtuzumab and immunoglobulin were recommended for the management of several steroid-refractory irAEs.

Although ICIs have revolutionized the current treatment and outlook for multiple cancer types, their therapeutic efficacy is restricted by irAEs. We have attempted to summarize the clinical and mechanistic features of irAEs and the related risk factors for their occurrence. We also generalized the mechanism-based therapeutic strategies to mitigate irAEs while maintaining therapeutic benefit of ICIs (Figure 2).

At present, the mechanism of irAEs is not fully elucidated, though tissue-specific irAEs are supposed to be meditated by T cells, B cells and a mixed aetiology. Recent development of murine irAEs models, including patient-derived xenograft and humanized mouse models, had a critical role for designing specific therapies and guiding precision medicine approaches in clinic (Shultz et al., 2019). Furthermore, novel insights from clinical and high-throughput data to develop accurate irAEs models contributed to understanding the etiology and pathogenesis of irAEs following immunotherapy, as well as prioritizing the clinical therapeutic regimens for clinicians (Bayless et al., 2021).

Currently, development of novel immunotherapeutic approaches may provide alternative treatments for irAEs. As the gut is a known modulator of specific T cell subsets (Gopalakrishnan et al., 2018), and the gut microbiome plays a role in driving autoimmunity and irAEs (McCulloch et al., 2022), altering the microbiome may be a promising strategy for irAEs. Wang et al. reported a successful treatment of ICI-related colitis through fecal microbiota transplant (Wang Y. et al., 2018). Another intriguing targeted immunotherapeutic approach is the use of bi-specific antibodies (binding to two or three different entities simultaneously). T cell-based bispecific antibodies could bridge T cells to tumor cells to facilitate more focal T cell activation (Labrijn et al., 2019), which might be a novel potential approach to abrogate the toxicity of ICIs while maintaining their therapeutic benefit.

Taken together, comprehensively understanding the clinical and mechanistic features of irAEs will be conducive to taking mechanism-based measures in clinical practice and thus providing the crucial help for clinicians to deal with them.

## Abbreviations

ICIs, Immune checkpoint inhibitors; irAEs, immune-related adverse events; CTLA-4, cytotoxic T lymphocyte protein 4; PD-1, programmed cell death 1; PD-L1, programmed cell death ligand 1; ASCO, American Society of Clinical Oncology; NCCN, National Comprehensive Cancer Network; SITC, Society for Immunotherapy of Cancer; ESMO, European Society for Medical Oncology; CTCAE, Common Terminology Criteria for Adverse Events; NSCLC, non-small-cell lung cancer; IBD, inflammatory bowel disease; WBC, white blood cell; RLC, relative lymphocyte count; NLR, neutrophil-to-lymphocyte ratio; PLR, platelet-to-lymphocyte ratio; Treg, regulatory T; Th17, type 17 T helper; BP, bullous pemphigoid; TNF, tumor necrosis factor; FNγ, interferon-γ.
